# Effects of Vaccination against Pandemic (H1N1) 2009 among Japanese Children

**DOI:** 10.3201/eid1704.100525

**Published:** 2011-04

**Authors:** Hiroshi Nishiura, Hitoshi Oshitani

**Affiliations:** Author affiliations: Japan Science and Technology Agency, Saitama, Japan (H. Nishiura); University of Utrecht, Utrecht, the Netherlands (H. Nishiura); and Tohoku University Graduate School of Medicine, Sendai, Japan (H. Oshitani)

**Keywords:** Viruses, influenza, children, Japan, vaccination, inactivated vaccine, pandemic, pandemic (H1N1) 2009, Japan, letter

**To the Editor**: We report findings from a household-based study on the protective effects of vaccination against pandemic (H1N1) 2009 among Japanese children. In Japan, prioritized vaccination started in October 2009, focusing on health care workers, pregnant women, persons with underlying diseases, and children 1–9 years of age. Only nonadjuvant split vaccines (inactivated) produced by 4 manufacturers (Denka Seiken, Tokyo, Japan; Kaketsuken, Kumamoto-shi, Japan; Kitasato, Tokyo, Japan; and Biken, Suita-shi, Japan) were used by the end of January 2010 (*1*). Because the protective effects of vaccination at the individual level are best measured by household data (*2*), we conducted a retrospective household survey involving 1,614 nonrandomly sampled households (i.e., based on area sampling of households across Japan, according to the regional population size, with a total of 6,356 household members), in which the earliest cases were diagnosed from October 2009 to mid-February 2010. Our study aimed to assess vaccine-induced reductions in susceptibility and infectiousness among children by using the household secondary attack rate.

Influenza cases were defined as confirmed cases (i.e., diagnosed by real-time PCR) or influenza-like illness (ILI) cases (i.e., in febrile patients [>37.5°C] with cough and/or sore throat). The cases had to meet the following inclusion criteria for analyses: 1) index case-patient and exposed persons in households were healthy children 1–9 years of age (households with <2 children were excluded), because age-specific susceptibility and infectiousness can greatly influence the frequency of household transmission (*3**–**6*); b) all exposed persons shared the same household with index case-patients for at least 1 of 7 days after illness onset of the index case-patient; c) index case-patient did not receive treatment with antiviral agents (e.g., zanamivir or oseltamivir) within 2 days after illness onset; d) time interval from illness onset of the index case-patient to that of subsequent case-patients was <7 days (*7**,**8*); and e) vaccinated persons received their first vaccination >28 days before illness onset (if index case-patient) or exposure (if not index case- patient).

In total, 251 children met the above criteria, comprising 109 index case-patients and 133 unvaccinated and 9 vaccinated exposed persons. The mean age was 6.4 ± 2.1 SD years. Among the 251 children, 15 (6.0%) had been vaccinated, and 169 (67.3%) had received a diagnosis of influenza. Confirmed cases accounted for 17.8% (30/169) of cases; 21 patients were the index case-patients in individual households. The mean age of patients with confirmed diagnoses was 6.5 ± 2.0 SD years and did not differ significantly from the ILI patients.

Let SAR*_ij_* represent the household secondary attack rate (SAR) with vaccination statuses of the index patient *j* and exposed persons *i* (where *i* or *j* is 0 or 1 for unvaccinated or vaccinated, respectively), and let *b* represent both groups. Among 133 exposed unvaccinated children, ILI developed in 59, yielding an SAR_0_*_b_* of 44.4%. Among 9 exposed vaccinated children, ILI developed in 1 child, yielding an SAR_1_*_b_* of 11.1%. The difference between these SARs was marginally significant (p = 0.08 by Fisher exact test), and the susceptibility reduction was 1 – SAR_1_*_b_*/SAR_0_*_b_* = 75.0% (95% confidence interval [CI] –60.5% to 96.1%). Considering only exposures caused by unvaccinated first patients, SAR_00_ and SAR_10_ were 44.7% (59/132) and 0% (0/4), respectively. When the first patients with ILI in households were unvaccinated, ILI was observed in 59 of 136 children, yielding an SAR*_b_*_0_ of 43.4%. Among 6 exposures caused by vaccinated first patients, ILI developed in 1 person, yielding an SAR*_b_*_1_ of 16.7%. Although not significant (p = 0.40), the reduction in infectiousness by vaccination was estimated to be 1 – SAR*_b_*_1_/SAR*_b_*_0_ = 61.6% (95% CI –132.3% to 93.6%). The SAR_01_ was 0% (i.e., 1 exposure to an unvaccinated person caused by a vaccinated first patient did not result in influenza). Limiting the definition of influenza to confirmed cases, all 8 exposures to vaccinated persons did not result in influenza, and SAR_0_*_b_* and SAR_1_*_b_* were 10.8% and 0%, respectively. Similarly, all 5 exposures caused by vaccinated first patients did not result in confirmed cases, and SAR*_b_*_0_ and SAR*_b_*_1_ were 10.5% and 0%, respectively.

Although the CIs of the estimates included zero because of the small sample size, the expected reductions in susceptibility and infectiousness were 75.0% and 61.6%, respectively, which is consistent with findings from a meta-analysis of vaccine efficacy against seasonal influenza (*9*). Two limitations must be noted, namely, estimates based on nonrandom samples and a case definition that relied on symptoms of case-patients. The former point cannot be explicitly addressed by a retrospective study design, but we enforced strict inclusion criteria for analyses and limited our study to healthy children. Accounting for the latter point (e.g., serologic diagnosis to capture symptomatic and asymptomatic cases) could yield slightly higher estimates than ours, provided that vaccination reduces the probability of clinical illness if infection occurs. Thus, despite these limitations and a critical need for further studies that include estimations of effectiveness (*10*), our results provide insight into the effects of vaccination in reducing risks for infection and clinical attack among children exposed to pandemic (H1N1) 2009 virus in their households.
